# Strategies for maintaining a positive body image in the face of stigma

**DOI:** 10.1177/13591053251330433

**Published:** 2025-07-19

**Authors:** Bridget Dibb, Erica G Hepper, Sarah-Jane Stewart, Menna Price, Laura L Wilkinson

**Affiliations:** 1 University of Surrey, UK; 2 University College London, UK; 3 Swansea University, UK

**Keywords:** coping strategies, obesity, positive body image, qualitative research, weight stigma

## Abstract

This study aimed to explore how individuals with obesity, who have a positive body image (an accepting and favourable view of the body), maintain this positive image in the face of weight stigma. A qualitative methodology was used to explore the experiences of 16 individuals (11 women; 5 men) using one-to-one semi-structured interviews. Four themes were developed using Thematic Analysis: Experiences of stigma, Self-evaluative cognitive strategies, Social Behavioural strategies and What Helps? Themes included strategies that reflect self-affirmation, defensive self-protection and social support. The results also included strategies not found in previous research, such as confronting the source of the stigmatisation. Moreover, no negative strategies, such as eating or self-harm, were reported. In addition, the participants spoke of the positive effect of social support but did not report seeking this support, which suggests that perceived social support may be more useful than enacted support.

## Introduction

While living with obesity can directly impact physical health and put the individual at risk of several chronic conditions (including diabetes, coronary heart disease and more) it can also affect mental health, where individuals may experience stigma, a negative body image and depressive symptoms. However, not all people living with obesity have a negative body image; some people who live with obesity maintain a positive body image and do not appear to be as affected by the negative experience of stigma. Less is known about how this group maintain their positive body image in the face of stigma. This paper explores this question using interviews with men and women who experience both obesity and a positive body image.

The rates of obesity and overweight are estimated to be at 2.5 billion worldwide and rising (WHO, 2024). Mirroring this is the rise in the experience of weight-based stigma (Myers and Rosen, 1999; Puhl et al., 2007, Puhl and Heuer, 2009; Westbury et al., 2023). This is concerning due to the consistent evidence base detailing the relationship between experiencing weight stigma and poorer health outcomes (for a review see Papadopoulos and Brennan, 2015). Mounting evidence suggests that this effect is partly driven by individuals internalising the stigma (Puhl et al., 2007). Internalised stigma comprises the following key features: ‘i) awareness of negative stereotypes about one’s social identity; ii) agreement with these stereotypes; iii) application of these stereotypes to oneself; and iv) self-devaluation due to one’s social identity’ (Corrigan et al., 2006). Internalised weight stigma specifically is associated with increased depression, anxiety and psychological distress as demonstrated in a systematic review and meta-analysis of 30 studies (Alimoradi et al., 2020). The consequences of stigma also impact physical health and weight management.

This association is accounted for in the Cyclic Obesity/Weight-Based Stigma model (COBWEBS), in which weight stigma is seen as a stressor or catalyst for stress (increased cortisol), leading to increased risk behaviours (eating behaviour) and, ultimately, weight gain (Tomiyama, 2014). Given that internalised weight bias mediates the effects of experienced weight stigma on stress, it is important to understand how some individuals can be more resilient to experiences of stigma and avoid internalising them. The experiences and perceptions of weight stigma for those who hold a positive body image may provide important clues (Schwartz and Brownell, 2004).

Research exploring the coping mechanisms used by those with obesity experiencing weight stigma highlights a range of strategies (Cash et al., 2005; Gerend et al., 2021; Myers and Rosen, 1999; Puhl and Brownell, 2003). Myers and Rosen’s (1999) early study of coded questionnaire descriptions indicated that ways of coping with obesity stigma included positive ‘self-talk’, countering stigmatising remarks, using faith, religion or prayer, eating and positive reframing. This was supported by a review which showed many ways of coping, including attempts to deal with and change the situation, and feelings of pride (Puhl and Brownell, 2003). Further work was facilitated by the development of a scale measuring coping with body image threat (the Body Image Coping Strategy Inventory; Cash et al., 2005), which identified three areas of coping: avoidance (of situations where stigma may be experienced), appearance fixing (covering or changing one’s appearance), and positive rational acceptance (acceptance and positive thoughts about one’s appearance). A more recent qualitative study (Gerend et al., 2021) found 12 coping strategies among individuals who experienced obesity stigma. The strategies supported those included in the Body Image Coping Strategy Inventory but also uncovered some new strategies, such as eating, distraction, meditation, social withdrawal, self-harm and rumination. Inspection of the range of coping strategies for this sort of stigma indicates that individuals can use varied strategies. Importantly, the helpfulness or effectiveness of these strategies also varies. Whereas some are perceived by participants as helpful or effective, other strategies were recognised as not useful (Gerend et al., 2021) or are known to contribute to poor mental health or physical risks (e.g. comfort eating, rumination, self-harm).

While the above studies discussed the coping strategies of people living with obesity, it is unclear what strategies are used by people with obesity who hold a positive body image (Schwartz and Brownell, 2004). A positive body image has been described as ‘accepting and holding favourable opinions towards and respecting the body’ (Tylka and Wood-Barcalow, 2015: 53) and people who hold a positive body image may have different experiences and/or use different coping strategies in the face of stigma. Knowledge of these coping strategies to maintain a positive stance, and which protect body image in the face of stigma, may be applicable and useful for others who struggle to hold a positive body image (Cwynar-Horta, 2016). In support of this, research has shown that exposure to those who are average-sized or body positive (those who have a and hold a positive body image) can have a positive effect for those living with obesity, leading to increases in emotional and physical wellbeing (Andrew et al., 2016; Cohen et al., 2019; Linardon et al., 2022) and reduced negative mental health outcomes (for a review Linardon et al., 2022). Intervention studies show that those who were either exposed to positive body images (Cohen et al., 2019; Stevens and Griffiths, 2020) or who listened to positive body-related songs (Coyne et al., 2021) reported increased satisfaction with body size and positive mood. This suggests that body positive images and environments may counter the negative effects of obesity stigma. Nevertheless, individuals with a positive body image are also likely exposed to images and environments that perpetuate weight stigma, and they may respond to such experiences in particular ways that help to maintain their positive body image.

Existing research exploring weight stigma coping strategies offers useful insights into how individuals handle such experiences, but to date there is no research focusing specifically on the strategies used by individuals with obesity who hold a positive body image. Knowledge of the strategies used by this group specifically would provide vital insights into methods to protect an individual’s sense of self and body image and provide guidance for the focus of interventions that aim to reduce the damaging impacts of weight stigma. It is also important to explore what types of stigmatising experiences, if any, individuals with a positive body image face. If this group turns out not to be exposed to weight stigma, then this could explain how they maintain a positive body image. However, if this group faces similar weight stigma experiences to those documented in past research, then their perceptions and reactions to these experiences may hold vital clues to how they maintain a positive body image. To this end, this study aimed to purposively recruit and interview individuals with obesity who self-identify as having a positive body image to explore qualitatively how they maintain this positive body image in stigmatising situations.

## Methods

### Participants

Sixteen participants were recruited from social media sites (e.g. Facebook, Twitter, LinkedIn, Instagram) and from the undergraduate cohort of a British University. Inclusion criteria ensured participants were UK residents over 18 years of age, with a BMI over 25 kg/m^2^, who self-identified as having a positive body image. Specifically, we recruited those who self-identified as having a positive body image and used a score of >30 on the Body Appreciation Scale as an indicator that they did not hold a neutral or negative body image (Tylka and Wood-Barcalow, 2015). Participants (Table 1) included more women (*n* = 11; 69%) than men (*n* = 5; 31%), with an age range of 19–56 (*M* = 31.19, SD = 10.90). Just over half the sample (*n* = 9) identified as white. The final sample BMI ranged from 28 to 43 kg/m^2^ (*M* = 31.88, SD = 3.85) and scores on the Body Appreciation Scale (BAS; (Tylka and Wood-Barcalow, 2015) ranged from 30 to 50 (*M* = 41.56, SD = 6.03). Participants were excluded if they reported diagnoses of eating disorders, other mental health disorders or identified as having a physical disability (due to possible experiences of other stigma).

**Table 1.** Participant demographic information.

Participant number	Sex	Ethnicity	Age	BAS score	BMI
1	F	White	39	49	35
2	F	White	29	50	28
3	F	White	20	47	35
4	F	White	43	45	31
5	F	White	56	41	43
6	F	White	19	40	34
7	M	White	33	32	30
8	F	Mixed	29	44	31
9	M	White & Black African	24	41	30
10	F	White	33	32	32
11	M	White & Black Caribbean	24	43	31
12	M	White & Black Caribbean	23	41	30
13	M	White	19	30	29
14	F	Pakistani	24	48	28
15	F	Spanish	36	43	35
16	F	Black African	48	39	28

BAS: Body Appreciation Scale.

### Procedure

After receiving ethical approval from the University’s Ethics Committee, adverts were posted on social media, and posters were placed in public areas such as bus stops, public noticeboards, and shop windows. Students were also recruited using the University’s research participation scheme. Email recruitment took place via ‘Plus Size social events’ Meetup group. Snowball sampling was also used to encourage sharing the advert to others.

After consenting, participants completed a screening survey (hosted on Qualtrics), where they reported their demographic information (gender, age, height and weight). They then completed the Body Appreciation Scale 2 (Tylka and Wood-Barcalow, 2015) to assess positive body image (10 items, e.g. ‘I respect my body’, ‘I feel like I am beautiful even if I am different from media images of attractive people e.g. models, actresses/actors’). Participants rated the extent to which the item was true (1 = never, 5 = always). Participants whose average score exceeded the midpoint (i.e. sum > 30) were eligible.

Eligible participants were interviewed online, via Teams or Zoom, to allow recruitment from a wider pool of participants (Lobe et al., 2022); and reports show there is little difference in the quality of the data from online versus in-person studies (Namey et al., 2020, 2022). Consent was re-affirmed via an online form at the time of data collection. The mean duration of interviews was 31 minutes (range 18–71 minutes). All interviews were recorded, anonymised and transcribed for analysis (the transcripts were not returned to the participants after transcription following the guidance of Hagens et al. (2009), as the benefit of doing this is limited and it can be burdensome for the participants who have already given up their time for the interview).

### Interview schedule

Data collection took place using semi-structured interviews, which allow the researcher to build a good rapport with the participant and facilitates in-depth discussion. The design of the interview schedule followed a participant-led approach, using open questioning, to encourage discussion. The interview schedule was pilot-tested with one individual; data from this interview are not included in the results. The interview began with a broad question, ‘I am interested in what you feel about your own body size. Can I start by asking you to tell me how you feel about your body size and shape?’, to encourage discussion on points the participants felt were important. To explore experiences in social situations, where stigmatising experiences may occur, further questions asked more specifically about (a) whether other people notice or comment on the participant’s body size and (b) how they think body size and weight are portrayed in the media. After each of these, follow-up questions asked how the participant *feels* when they experience that, what the participant *thinks* when they experience that, and how they deal with it. Subsequently, participants were invited to talk about how they maintain positive or comfortable views of their body size. Prompts and follow-up questions allowed a deeper discussion of the participants’ responses. Finally, the interview ended with a mood repair question about the most positive or happy thing in the participant’s life.

### Data analysis strategy

Thematic Analysis was carried out using the steps proposed by Braun and Clarke (2006), comprising an iterative process of initial coding, collating and grouping of codes into initial and then final themes. The analysis and theme development occurred inductively, that is, using a bottom-up approach, in order to explore experiences without being restricted by any theoretical framework. All analysis was carried out by BD and SJS, both experienced in qualitative analysis. Experiences of weight stigma were coded to understand whether people who maintain body positivity still face stigmatising experiences or not. Strategies for maintaining body positivity were coded both from participants’ explicit description of such strategies (e.g. ways that they told us they tried to respond) and also from the way that participants spoke about their experiences and responses (e.g. phrases that they used naturally when describing their experiences, without labelling them as strategies).

## Results

The main aim of the study was to explore how individuals with obesity who hold a positive body image experience stigmatising situations and how they maintain positive body attitudes in these situations. While the participants spoke of many experiences regarding their body size, the themes below (Table 2) were developed specifically from the comments that focused on, or reflected, strategies they used to maintain resilience against weight stigma. Theme 1 gives examples of the sort of negative interactions participants have experienced, including with friends and family, in the media or society in general. Theme 2 was developed from reports of how participants coped with and maintained a positive view of themselves using self-evaluative strategies that embraced or transcended their size (subtheme 2.1) or that ignored or dismissed stigmatising comments (subtheme 2.2). Theme 3 presents evidence of a more behavioural response where participants confronted or physically avoided the stigma. Finally, Theme 4 was developed from comments that highlight what participants found helpful.

**Table 2.** Overview of themes and subthemes.

Theme	Subtheme
Theme 1: Stigmatising experiences	1.1 Interactions with others 1.2 Society and Social Media
Theme 2: Cognitive self-evaluative strategies	2.1 Self-affirmation Strategies: ‘There’s so much more to me’ 2.2 Self-protection Strategies: ‘Don’t listen to other people’
Theme 3: Social behavioural strategies	3.1 Confronting Strategies: ‘How dare you say something like that’ 3.2 Avoiding Strategies: ‘I don’t want to even be near anyone that would be ashamed of me’
Theme 4: What helps? – the positive role of others	4.1 Positive support from others: ‘Having close friends who will stick by you through thick and thin’ 4.2 Positive Media: ‘Advertisements and such, they are a lot more realistic … they represent the real struggles of everyday life’

### Theme 1: Stigmatising experiences

This theme presents the data showing participants’ perceptions of how others viewed their size, which gave reason to why the participants needed to develop strategies for dealing with experiences of weight stigma. These experiences were described as both comments or actions by other people, including social media, and perceived societal attitudes, which were seen as underlying these comments and actions. These were largely negative and left the person feeling worse-off, indicating that people with both positive body image and obesity do experience weight stigma. Two subthemes were developed: 1.1 Interactions with others and 1.2 Social Media and Societal Attitudes.

#### Subtheme 1.1: Interactions with others

Participants described interactions with family members, partners and colleagues in which negative comments were made about their size and they were left feeling negative about themselves. Participant 4, below, describes a situation where she felt upset after her parents tried to offer her money as an incentive to lose weight,

And my dad offered me money to lose weight […] I was really upset because I was like […] it was just upsetting because they felt so strongly that they had obviously had this conversation and decided that this was going to work. *4* (F)

Below, Participant 3 describes how her negative attitude to ‘being fat’ was developed, as a child, through interactions with her family. The long-term and pervasive influence is clear as she describes how these attitudes were passed down over three generations,

My grandma said to my parents that they needed to have a conversation with me about that. My dad […] said to me, “You don’t want to grow up and be fat because you won’t find a boy that will fancy you and all the girls won’t want to be your friend. You’ll be a minger.” I was like, at seven, “Oh, alright then. […] So […], I’ve always been told that fat is bad”*3* (F)

These stigmatising interactions also occurred within romantic relationships. Participant 1 describes how ashamed she felt when her partner asked her to cover up her tummy,

Yes […] it’s horrendous, absolutely horrendous […] It made me feel just … horrible. It’s like you just want to crawl out of yourself. Yes, it’s horrible. […] So, say if [..] I reached up and my T-shirt rode up […], so I might expose a tiny bit of skin on my tummy, he’d be like, “Pull it down. Pull it down,” like I should be ashamed of that. Yes […], when I think about that, it was, it makes you feel horrendous and it takes away your confidence. *1* (F)

Colleagues were also the source of negative comments. Participant 8 shows how she perceived her colleagues to attribute negative stereotypes, such as laziness, to people with overweight,

Yes, just talking to colleagues, […] some people think that it’s just pure lazy people who have no self-control of what they eat, and that it’s a real bad characteristic to have. 8 (F)

#### Subtheme 1.2: Society and social media

Participants felt that social media supported the general view of the thin ideal with the sort of posts that are common, yet the negative effects of these posts are not considered,

the media has a huge impact. I think it has a negative impact […] because it portrays such a slim version of what a beauty ideal is. 2 (F)

Participants also expressed their perceptions of a societal pressure to conform to a thinner body. Participant 13 says that social media ‘makes people think they have to be a certain way’ which hints at the role that social media may have in the internalisation of the social perceptions,

It portrays a bad image because it makes people think they have to be a certain way to do certain things when you can be any shape and size and do absolutely anything. 13 (M)

Social media was mentioned by most participants in a negative way. Participants felt people on social media were hurtful with their comments as people feel they have permission to criticise others about their appearance,

Sometimes when being exposed to social media things [or] TV shows […] like, “My 600-lb Life” or whatever […] I’ll kind of feel a bit down that people are profiting off that sort of thing. 14 (F)

### Theme 2: Self-evaluative cognitive strategies

This theme encapsulates participants’ cognitions with regard to maintaining their positive body attitude, resisting internalising societal views and managing their negative interactions with others (described in Theme 1). Two types of cognitive strategies were identified. The first subtheme, *Self-affirmation Strategies*, includes strategies in which participants accepted and embraced their size and took the view that there is more to life than weight. The second subtheme includes cognitions that helped participants to dismiss their negative experiences (*Self-protection Strategies*), for example ignoring comments and controlling negative thoughts.

#### Subtheme 2.1: Self-affirmation strategies: ‘there’s so much more to me’

In the face of the negative experiences described in Theme 1, participants spoke of cognitive strategies that they used to manage and maintain their positive frame of mind about themselves. These strategies ranged from accepting their size, to reinterpreting their size to focus on their physical health, to reminding themselves that there is more to life than weight. These positive cognitive strategies illustrate aspects of self-acceptance and self-affirmation.

Some spoke of the importance of accepting themselves. Participant 15’s comment below indicates that she views accepting the way she is as important, and hints that this is the morally better approach to take.

the main thing is trying to accept yourself trying to find what is good in you and show that [you are] more than your flaws. 15 (F)

Similarly, Participant 9’s example of the need for self-acceptance in order to reduce conflict with others,

you just have to accept who you are at the at the moment, because if you really if you don’t accept yourself, you start fighting with people […] you can exchange blows. 9 (M)

In a similar vein, many participants also indicated a shift in their values in order to view themselves positively and affirm their worth. Participant 12 reminded themselves that they are the same as others,

We have a life to live in and we have a life to you. And we are, we are people like any other like any other. So that spirit is what drives me forwards. 12 (M)

The comments below show how participants felt they ‘were more than my body’ or that their ‘body didn’t define them’. Participant 2, below, speaks of how they clearly valued their other important life roles, and this enabled them to not focus on their weight or size.

I started to develop and started to see that I was more than my body, and see that I was a kind, caring friend. I was a girlfriend. I was a daughter, a sister, and I started to care less about the way that I looked 2 (F)

The shift in focus described above was also evident in comments that emphasised that health is more important than appearance. Participant 1’s compensatory beliefs are evident in the way she talks about how her body works,

I think I look alright. I feel alright. Everything works. I generally feel attractive, it’s not… That’s okay. Yes, there’s more to worry about than having the perfect body, if you know what I mean, so, yes, I guess 1 (F)

#### Subtheme 2.2: Self-protective strategies: ‘Don’t listen to other people’

This subtheme shows how participants engaged in self-protective strategies by ignoring threatening comments, or negating them or by criticising the source, indicating a more defensive response compared to subtheme 2.1. Participant 2 advocates not listening to others and speaks positively about how to feel about one’s body,

Don’t listen to other people, […] You know what you know. Do your research on your body size. Don’t let anybody else tell you how you feel about your body. You have a right to be in a bigger body because at the end of the day we’re all unique and all individual 2 (F)

Ignoring also took the form of avoiding thinking about the topic altogether. Participant 15 talks about stopping the thoughts and trying to ‘do something different’, showing both an attempt to control her thoughts and to distract herself,

So just try to stop the thinking. Try to do something different. Try to um. Whatever. If it’s something that you can do something about it, then do it. Don’t just to leave it. Do it- 15 (F)

While ignoring strategies were common, rationalising was also used to comfort themselves that they would not see the person again. This also highlights the reassurance that they give themselves by talking to themselves,

when I’m walking past someone I just have to mentally be like, “Okay, they’re only going to be staring for another *two* seconds, I’m never going to meet this person again. It shouldn’t matter so it’s not allowed to affect me, I’m not going to let it. 6 (F)

A defensive approach was also taken with the media, where participants objected to the negative view of overweight and obesity. Both participants below give examples of how they viewed unwanted social media that targeted them, as fake and this allowed them to disregard the information.

The keto tablets that will magically make you lose weight. People should know that most, I mean all, most of them are scams and it’s just they’re all lies basically 5 (F)

… sometimes sponsored adverts come up when you like don’t want them or they just they pop up regardless of […] what you actually wanna see, they pop up anyway to promote their brands and it’s it’s annoying to see. But […] I scroll and it’s easier to think now it’s fake 13 (M)

### Theme 3: Social behavioural strategies

This theme was developed from comments that indicated that the participant acted on their feelings in response to stigmatising experiences. Two subthemes emerged that referred to *confronting* (approach-oriented) and *avoiding* (avoidance-oriented) behavioural responses.

#### Confronting Strategies: ‘How dare you say something like that’

Many participants advocated confronting the source of the stigmatisation. Participant 10 felt the best way of dealing with it is to take the first step and talk about it before anyone else does,

It’s about kind of bringing it up in a way that I feel comfortable with before someone else can omit, can deal with it. I think it helps other people know that. Kind of it’s OK to talk about in a in a certain way. 10 (F)

Participant 3 below show they are quite comfortable confronting their mother,

I have had to say extreme things […] ‘I will not come and see you. I will not speak to you until you change the way that you speak to me and about me’. And I don’t want to come to that, and I don’t think I will, but I have to threaten her as a way of saying, ‘Stop it’. 3 (F)

Others felt that sometimes it took them a moment to respond or they had to work up the courage to confront others, showing that this type of response was effortful,

I think I had a few minutes to think about it and I was like, “How dare you. How dare you say something like that? I’m just really glad that you don’t get to share my body, because you don’t deserve that if you’re going to… ” 1 (F)

#### Avoiding strategies: ‘I don’t want to even be near anyone that would be ashamed of me’

This behavioural subtheme links well to the cognitive strategy of dismissing or self-protection (Theme 2.2), which showed how participants cognitively shut themselves away from stigmatising messages. Participant 11’s quote below is a good example of how they did not just ignore the comment but they walked away so that they could actually avoid the situation,

sometimes I try to walk away so that you showed them I don’t care about what they think, but deep down I know [..] I know it hurts. Because […] nobody wants people talking behind your back […] It just makes you uncomfortable 11 (M)

Note that for Participant 11, this behaviour was not just for his own benefit, as he feels this behaviour will communicate to the other person that he doesn’t care. Others felt that avoidance was more of a self-protective strategy,

I’ll only date people that are happy with my body and stuff. I think the reason I do that is because I don’t want to even be near anyone that would be ashamed of me, or against my size because it just makes me very uncomfortable and rejected really. 3 (F)

While avoiding can be self-protective, avoiding situations to avoid anticipated stigma can lead to participants missing out on important interactions with others, as participant 6 demonstrates,

It’s been a while since I’ve been to a family gathering, because of these issues I tend to avoid them nowadays 6 (F)

### Theme 4: What helps? – The positive role of others

This theme was developed largely from the interview questions about what participants felt to be helpful. The emerging theme is less about strategies that participants use themselves, but more about support received from external sources.

#### Positive support from others: ‘Having close friends who will stick by you through thick and thin’

This subtheme focuses on how the influence of other people’s behaviours and comments can support those who are comfortable with their weight. Some participants expressed the positive feeling of being accepted by others,

…have people around you. Yeah. Yeah. Friends and families they […] Yeah, very important thing. You don’t need to pay anything. You just get free advice or something. Yeah, friends and family 16 (F)

having supportive friends and family, I think that is a positive thing and also having close friends who will stick by you through thick and thin.. *9* (M)

Others talked about feeling positive after receiving compliments,

The woman that was there that was fitting me, she was like, “I’m so envious of your body. You’ve got the perfect hourglass curve thing going on.” I was like, “Oh, thank you.” So in some ways it can be really nice. 3 (F)

I suppose because I have broader shoulders and, like I said, I’m quite a big guy generally. If I do stand next to other people who might be similar, it could be guys, for instance or something, they always notice or compliment me on my physique. Saying, “Oh, you’re so much bigger than I am. I wish I was your size.” So yes, it’s not just women, it’s also guys in a complimentary way. 7 (M)

#### Positive Media: ‘Advertisements and such, they are a lot more realistic … they represent the real struggles of everyday life’

There was also a place for the media in this theme. Participants were sensitive to how overweight and obesity are portrayed in the media (evidenced in Theme 1) but were equally appreciative when they felt recognised and treated fairly, showing that it can play a positive role,

In most modern media, at the moment, they’re doing a really good job of making plus size characters and people just be people. Being plus size or fat, for the use of the descriptor word, that isn’t their character trait. They’re just a character who happens to be plus size, that is fantastic. 6 (F)

## Discussion

This study aimed to understand the experiences of individuals with obesity who maintain a positive body image and how they maintain this positive view in the face of weight stigma. It was possible that people with a positive body image do not experience weight stigma and this may account for their positive attitude, but our results confirm that this group do indeed still experience weight stigma. This occurred both with their own personal interactions and interactions on social media, which left them feeling negative and feeling the need to conform to a thinner body shape. This links well to the evidence of weight stigma experienced by those who do not specifically hold a positive body image (Papadopoulos and Brennan, 2015) and so shows that people with a positive body image appear to experience the same type of weight stigma as others. Accordingly, the strategies used by those with a positive body image in response to these experiences may be crucial to understanding their resilience.

Our results show how people with a positive body image maintain their positive attitude in the face of a society where weight stigma is common. The self-evaluative cognitive strategies that our participants used included self-affirming strategies and self-protective strategies. The self-affirming strategies included an acceptance of their size and a shifting of their values and priorities, for example, to see that they are more than their ‘flaws’ or that there are more important aspects of life than body size. Self-affirmation is a means of bolstering the self by focusing on values, strengths or positive experiences in other domains (Steele, 1988). Self-affirmation is well-established as a way to preserve feelings of self-integrity, self-worth and wellbeing in the face of threats (e.g. feedback, health messages, stress; Cohen and Sherman, 2014; Howell, 2017). It can be conceptualised as a form of self-enhancement (Hepper et al., 2010) or a more adaptive form of self-protection that enables one to process a threat in a less defensive way (Hepper et al., 2022; Sherman and Hartson, 2011). Self-affirming strategies have previously been reported by interviewees as ways of coping with weight stigma (Alleva et al., 2023; Gerend et al., 2021) and divorce (Butkutė et al., 2023). Amongst other coping responses used by participants with obesity in prior literature, our self-evaluative cognitive strategies align with self-talk and acceptance (Myers and Rosen, 1999) and positive rational acceptance, where individuals coach themselves about their positive attributes (Cash et al., 2005; Tylka and Wood-Barcalow, 2015). Our evidence shows for the first time that people with a positive body image, in particular, make use of these strategies. Promisingly, there is evidence that self-affirmation may lead to weight loss, if desired (Logel and Cohen, 2012). This is also supported by prior research that has shown that increasing body diversity in the media relates to improved beliefs surrounding functionality satisfaction, in which the focus is instead on what a body is capable of, rather than what it looks like (Mulgrew and Tiggemann, 2018).

The more dismissive cognitive strategies that emerged in our study included not listening to what other people have to say, ignoring stigmatising messages, or criticising the source, whether it be a person or a media-based message. These all reflect defensive self-protection strategies, which are intended to deflect and protect self-worth from threats (Hepper et al., 2010). There was also evidence of participants using distraction techniques, which are also self-protective behaviours (Gerend et al., 2021; Myers and Rosen, 1999). These comments tended be phrased in a way that indicates a positive, optimistic attitude to weight. Along with the positive, self-affirming cognitive strategies, these self-protective strategies might serve to reduce aspects of internalised stigma (cf. Corrigan et al., 2006). That is, although participants reported awareness of negative stereotypes, self-affirmation may help to reduce agreement with them, defensive self-protection may help to reduce application of stereotypes to the self, and both may help to reduce resulting self-devaluation.

However, while self-protection strategies may function in protecting self-worth, they have the disadvantage of not allowing the individual to learn from the situation (Cohen and Sherman, 2014; Sherman and Cohen, 2006). In line with these cognitive signs of self-protection was the Social Behavioural theme in which participants described actively avoiding stigmatising situations or people. This is supported by previous research; avoidance has been widely reported as a common strategy amongst other studies exploring the stigmatising experiences of those with obesity (Cash et al., 2005; Gerend et al., 2021).

Another, more approach-oriented type of self-protection is confronting; our participants also spoke of confronting the source of the stigma. This usually involved speaking out to the friend or family member. This behaviour requires some confidence and aligns well with Myers and Rosen’s (1999) coping response inventory (being nice to others, educating others, and telling them off). However, this is not one of the main coping strategies reported by Cash et al. (2005), Gerend et al. (2021), or as a prevention strategy aimed at improving body image (Tylka and Wood-Barcalow, 2015). The participants in our study all reported having a positive body image and it is possible that these individuals felt empowered to confront those who make stigmatising comments or may have not internalised the stigma and felt confident to confront the source of the stigma. Interventions to help people cope with weight stigma may therefore benefit from a focus on self-efficacy to encourage or facilitate effective confrontation.

The final theme was developed from comments that related to what participants reported helped them in stigmatising situations (as opposed to how they themselves dealt with them). This theme related to external support, as comments all referred to family and friends’ unconditional support and acceptance as helpful. Participants also referred to the positive effects of the media where it was comforting to see large sized models used in advertising (Stevens and Griffiths, 2020). Self-enhancement was evident wherein participants felt good about themselves after these interactions (Sherman and Hartson, 2011). Seeking social support is one of the 12 coping strategies listed in prior research (Gerend et al., 2021; Myers and Rosen, 1999), so while our participants did not speak of seeking support, their comments still support the positive role that social support may play in dealing with weight stigma. Research shows that it is often the perception of available social support, more so than actual receipt of enacted support, that is most related to successful coping and wellbeing (e.g. Siedlecki et al., 2014). Indeed, sometimes receiving visible support can be harmful by implying weakness or indebtedness (Bolger et al., 2000). Hence, our participants may not have felt the need to seek social support actively even though they acknowledged finding it helpful, but further research is needed to confirm this. This suggests that interventions to help with weight stigma may focus on perceived availability of social support (rather than ‘actual’ enacted social support).

It is noteworthy that despite the prominence of supportive close relationships in participants’ accounts of what is helpful, some of their strategies for managing weight stigma involved confronting or avoiding close others—strategies that entail risk of damaging the very relationships that could be providing support. Perhaps individuals with a positive body image engage in ‘social pruning’ to focus on the relationships that already provide them with the support they need and move away from potentially toxic relationships. Future research could explore whether this selective narrowing of social networks serves emotion regulation, similar to that seen most strongly in older adulthood (O’Brien and Hess, 2020). However, several participants expressed regret that they were unable to have positive relationships with family members. Moreover, more constructive confrontation interactions could have the potential to instil change in the other person, cultivating an improved social support network and ultimately contributing to improved societal attitudes. Future research could explore the conversations and communication styles in such interactions to understand what creates a positive outcome for both parties.

While our results have supported some previous research, in comparison with the weight stigma coping strategies identified in the literature (Cash et al., 2005; Gerend et al., 2021; Myers and Rosen, 1999) we did not find that participants reported eating, meditation, self-harm, rumination, weight management, impression management (Gerend et al., 2021), use of faith or religion (Myers and Rosen, 1999) or appearance fixing (Cash et al., 2005) as strategies. The above three studies all recruited people with obesity; however, none of them reported having a positive body image. Many of these strategies suggest a negative view of their size and shape (eating, self-harm, and rumination) and imply attempts at conforming to the thin ideal (weight management, impression management and appearance fixing), which may account for their omission from our data given that our participants had embraced their size and shape. The evidence of the use of meditation to reduce the stress associated with the stigma (Gerend et al., 2021) and religion to consolidate (Myers and Rosen, 1999) as strategies may relate the internalisation of the negative stereotypes in these samples, while our participants, having a positive body image, may not have internalised such views. Once again, further research is needed to confirm this. Our results show enhancement, protective and affirmative strategies were perceived as helpful for our participants and this holds promise for interventions to increase self-compassion and self-affirmation (Logel and Cohen, 2012).

Taken collectively, our findings add to the notion that a positive body image may counter some of the negative effects of obesity stigma. One explanation for this could be that it protects against the internalisation of weight stigma experiences. Given that body dissatisfaction and internalisation of the thin-ideal are key aspects of internalised weight stigma, it is plausible that a positive body image could serve as a protective factor. Our findings identify cognitive and behavioural strategies that might serve as mechanisms for this protection.

Our study is not without limitations. Inherent in all qualitative studies is the limitation that the results and conclusions are reliant on the verbal fluency of our participants and these reports are the perceptions of these individuals. However, we were interested in these very perceptions and so a qualitative methodology was the most appropriate method to allow an in-depth exploration of how people feel and react in weight stigma situations. We took a reflexive approach in the data collection and analysis to ensure we were aware of our biases and the coding and theme development took place within the research team. In addition, although data saturation was achieved, the results of our study may be limited by our sampling strategy, whereby those not using social media or who were not associated with the university may have been different in some way and hold different views. We attempted to avoid this by recruiting in more than one location. However, we were not able to look at differences between individuals depending on where and how they were recruited. Another limitation lies with the definition and range of positive body image in our sample. We recruited those who self-identified as having a positive body image, and while we did use the BAS to ensure that participants did not hold a neutral or negative body image, some were more positive than others. Finally, our sample varied in cultural background and age, but older adults and men were under-represented. Our study did not aim to assess the effects of cultural beliefs as, although these influence body image perceptions (Schwartz and Brownell, 2004), we were interested in experiences and reactions relating to stigma situations. Future research should aim to recruit older participants and more men for a fuller understanding of the experiences and strategies used by different groups.

### Conclusion

The results of this study broaden our understanding of the experience of women and men with a positive body image when facing weight stigma. Self-affirming, self-enhancing and self-protective strategies were used to manage the experienced weight stigma. We found some of the strategies used by men and women with a positive body image to mirror those with obesity in other studies who do not hold a positive body image. However, men and women with a positive body image do not report engaging in negative strategies (such as self-harm, rumination and eating) and instead report using assertive strategies such as confronting. In addition, positive social support in the form of social media, friends and family can be self-enhancing. Future research should aim to focus on interventions aimed at reducing the effects of stigma with training to increase positive body image using self-enhancement, self-affirmative and self-protective strategies.
